# Joint control of seasonal timing and plant function types on drought responses of soil respiration in a semiarid grassland

**DOI:** 10.3389/fpls.2022.974418

**Published:** 2022-08-15

**Authors:** Ruyan Qian, Yanbin Hao, Linfeng Li, Zhenzhen Zheng, Fuqi Wen, Xiaoyong Cui, Yanfen Wang, Tong Zhao, Ziyang Tang, Jianqing Du, Kai Xue

**Affiliations:** ^1^College of Life Sciences, University of Chinese Academy of Sciences, Beijing, China; ^2^Yanshan Mountains Earth Critical Zone and Surface Flux Research Station, Chinese Academy of Sciences, University of Chinese Academy of Sciences, Beijing, China; ^3^College of Resources and Environment, University of Chinese Academy of Sciences, Beijing, China; ^4^School of Mathematical Sciences, University of Chinese Academy of Sciences, Beijing, China; ^5^The High School Affiliated to Renmin University of China, Beijing, China

**Keywords:** soil respiration, autotrophic respiration, heterotrophic respiration, climate extremes drought timing, function types, stability

## Abstract

Globally, droughts are the most widespread climate factor impacting carbon (C) cycling. However, as the second-largest terrestrial C flux, the responses of soil respiration (Rs) to extreme droughts co-regulated by seasonal timing and PFT (plant functional type) are still not well understood. Here, a manipulative extreme-duration drought experiment (consecutive 30 days without rainfall) was designed to address the importance of drought timing (early-, mid-, or late growing season) for Rs and its components (heterotrophic respiration (Rh) and autotrophic respiration (Ra)) under three PFT treatments (two graminoids, two shrubs, and their combination). The results suggested that regardless of PFT, the mid-drought had the greatest negative effects while early-drought overall had little effect on Rh and its dominated Rs. However, PFT treatments had significant effects on Rh and Rs in response to the late drought, which was PFT-dependence: reduction in shrubs and combination but not in graminoids. Path analysis suggested that the decrease in Rs and Rh under droughts was through low soil water content induced reduction in MBC and GPP. These findings demonstrate that responses of Rs to droughts depend on seasonal timing and communities. Future droughts with different seasonal timing and induced shifts in plant structure would bring large uncertainty in predicting C dynamics under climate changes.

## Introduction

In terrestrial ecosystems, carbon (C) fixed by plants or stored in the soil would release into the atmosphere in the form of CO_2_ (i.e., soil respiration, Rs), thus Rs is an important part of the C cycle and the main way that ecosystems return CO_2_ fixed by photosynthesis to the atmosphere (Wang et al., 2015). Rs includes two sources: heterotrophic or microbial respiration (Rh) and autotrophic or root respiration (Ra) (Hanson et al., 2000). Rs and its components were sensitive to changes in precipitation (Liu L. L. et al., 2016; Du et al., 2020), especially in water-limited semiarid grasslands (Thomey et al., 2011; Zhang et al., 2022). For example, extreme drought reduced Rs by strongly limiting photosynthetic substrate supply and microbial activities (Wang et al., 2013; Xu et al., 2019). However, the synchronous or asynchronous response of Rh and Ra in the face of droughts is not well understood (Huang et al., 2018; Sun et al., 2019). Mounting evidence suggested that the frequency and intensity of extreme drought would increase due to anthropogenic climate change during this century (Mallakpour and Villarini, 2016). Therefore, understanding the response patterns of Rs and especially its components to extreme drought is critical for the assessment of the ecosystem C cycle in the context of extreme climate events.

Drought events can occur throughout the year and changes in water availability caused by drought determine plant growth and carbon uptake and release (De Boeck et al., 2011; Wolf et al., 2016; Zeiter et al., 2016; Zhang et al., 2019c). Since the demand of plants for water varies seasonally, the response of ecosystems to droughts may vary with different seasonal drought timing. For example, spring and summer drought strongly regulate carbon flux season by limiting the plant canopy development and inhibiting rapid plant growth (De Boeck et al., 2011; D’Orangeville et al., 2018). Especially, seasonal variations in precipitation during the warm or dry season have a more significant impact on ecosystems due to the evaporative demand at the peak (Zeppel et al., 2014; Sun et al., 2016). In contrast, during the late growing season, plants approach to senescence and photosynthesis is reduced and late season droughts have little negative impact on productivity (Dietrich and Smith, 2016; Kannenberg et al., 2019). Collectively, these findings addressed that the response of multiple ecosystem attributes to drought with different seasonal timing were inconsistent, such as leaf photosynthesis, net ecosystems exchange, flowering phenology, and reproduction (Dietrich and Smith, 2016; Meng et al., 2019; Hahn et al., 2021; Li et al., 2022). Since processes are particularly associated with Rs (Song et al., 2012; Ru et al., 2018; Post et al., 2020), droughts that occur at different stages of the growing season are expected to have different effects on Rs. In recent years, the research on drought timing discussed mainly the comparison between drought treatment and ambient control (Tammy, 2014; Denton et al., 2017; Meng et al., 2019). Although these are valuable, such a method is affected by the seasonal cycle and interannual variation of precipitation (Li et al., 2022). Therefore, knowledge of how Rs and its components respond to drought with seasonal timing is very limited and how to distinguish the drought timing and drought intensity is essential.

A growing body of literature has described the importance of plant functional type (PFT) in regulating the process of carbon cycle, such as Rs (Johnson et al., 2011; Kuiper et al., 2014; Zhou et al., 2019). The responses to drought stress vary among different PFTs due to differences in phenology, biomass allocation, and rooting depth (Prevéy and Seastedt, 2014). For example, shrubs have a competitive advantage over graminoids in coping with drought stress, possibly because the deep root distribution of shrubs is conducive to absorbing nutrients and deep soil moisture (Liu et al., 2011; Wilson et al., 2018). Species richness can determine ecosystem stability, with species-rich communities generally being more stable to drought stress (Roscher et al., 2013; Elst et al., 2017), mainly depending on the dominant species. The effects of extreme drought events during important phenological periods of dominant species may be greater than those during other periods (Hovenden et al., 2014; Meng et al., 2019). However, little attention have been given to comparing the regulation of PFTs in response to seasonal droughts.

Changes in plant performance due to environmental disturbance may alter the amount of carbon (C) that plants can allocate underground (Connell et al., 2021). Plant regulated Rs through impacts on autotrophic respiration generated by root growth (Luo and Zhou, 2006; Zhang et al., 2021). Also, litter production and root exudates indirectly impact heterotrophic belowground respiration by altering soil microbial activity (Carbone et al., 2011; Zhao et al., 2016). Under disturbance, water, nutrients, and other resources are limited to access, leading to changes in species richness and community composition (Metcalfe et al., 2011; Xu et al., 2015); ultimately, it has a whole range of effects on Rs. However, a long-term drought experiment found that the effects of microbial activity inhibition and plant community adaptation on Rs offset each other, leading to no significant changes in Rs (Zhou et al., 2016). Therefore, the regulation of PFTs to Rs during seasonal drought stress is controversial (Welp et al., 2007; Estruch et al., 2020; Yan et al., 2021) and further research are urgently needed.

Here, to explore the response of Rs to drought, especially with different seasonal timing events and in various plant function types, we, respectively, imposed an extreme drought in the early-, mid-, and late stage of the growing season on three modeled plant communities (i.e., two grass species community, two shrub species community, and their combination community). Specifically, we tested the following three objectives: (i) Do drought effects on total soil respiration and its components vary among seasonal timing? (ii) Whether the response of soil respiration to drought can be determined by different plant function types? (iii) How seasonal timing and plant function types regulate the effects of drought on total respiration and its components?

## Materials and methods

### General situation

We conducted the study in a semiarid grassland at the Research Station of Animal Ecology (44°18′N, 116°45′E 1079m.a.s.l) in Inner Mongolia Autonomous Region, China. The mean annual temperature (1953-2012) of this region is −1.4°C and the mean annual precipitation is 350 mm with 80% of the rainfall received in the growing season (May to September) (Zhang et al., 2019b). Among average precipitation during the growing season, 75% is ecologically effective precipitation (recorded daily precipitation >3 mm during the growing season (Hao et al., 2017)). The grassland is dominated by the xeric rhizomatous grass (*Leymus chinensis*), needle grass (*Stipa grandis*), and perennial forb (*Medicago falcata*). Many other representative plants are widely distributed in the study area, which are of great importance. The soil in this area is classified mainly as chestnut, with 60% sand, 18% clay, and 17% silt (Hao et al., 2018).

### Experiment design

The effect of drought on the Rs joint control of seasonal timing and plant functional types (PFTs) was studied using a two-way split-plot experiment design, with drought treatment in the main plots and PFTs in the sub-plots, with three replications. Four drought treatments were set up in the main plots with three replicates: early-stage drought (DE, May-June), mid-stage drought (DM, July-August), late stage drought (DL, August-September) treatments, and ambient treatment as control (CK), respectively (the division of growing season, see Li et al. (2019)). The ambient control plots remained without rainfall manipulation and received ambient rainfall year-round. According to a ∼60-year record provided by The Xilin Gol League Meteorological Administration, we defined an extreme drought event as 30 consecutive days without effective precipitation during the growing season because the longest interval between two consecutive rainfall events was 30 days. The rain-out shelters were used to prevent natural rainfall in the plots to achieve experimental droughts; for details see Hao et al. (2017).

Each main plot was made up of three sub-plots, corresponding to three PFT treatments: Graminoids (G; *Leymus chinensis* and *Stipa grandis*), Shrubs (S; *Caragana microphylla* and *Artemisia frigida*), and their combination (Graminoid × Shrub: GS). These four widespread species selected were dominant local species. The experimental plant communities were established in May 2012 (Supplementary Figure 3). Every species had the same proportion in terms of the seed quality for all communities. The quality of seeds for the species in four-species communities was half of corresponding species in two-species communities. The seeds were evenly sown after being well blended. According to the previous phenological record, the height of Graminoids plots was about 22 cm and the total coverage was about 70%, of which *Stipa grandis* accounted for about 40% and *Leymus chinensis* about 20%. For Shrub plots, the community height and coverage were 10 cm and 80%, of which *Caragana microphylla* was about 15% and *Artemisia frigida* was about 65%. The height of Graminoid × Shrub plots was about 15 cm and the total coverage was about 75%, including 10% of *Leymus chinensis*, 20% of *Stipa grandis*, 15% of *Caragana microphylla*, and 30% of *Artemisia frigida* (photos could be found in Supplementary Figure 3). Each sub-plot had an area of 2m × 2m and 1 m intervals between sub-plots. Data were collected from the central square meter of each plot to avoid the edge effect. To prevent horizontal water transfer, the metal sheet was placed 40 cm deep around each sub-plot and inserted 10 cm into the soil. At the beginning of the study in May 2017, air temperature (HMP45C temperature probe; Vaisala, Woburn, MA, United States) and photosynthetic active radiation (LI-190SB quantum sensor; LI-COR, Inc., Lincoln, NE, United States) were compared to ensure no significant difference between the value measured under the shelter and that measured at the open space near the plot. Throughout the experiment, the composition of the plant community was maintained by monthly removing seedlings of all other species.

### Soil respiration and microclimate measurements

The modified Mesh-bag method was used to distinguish Ra and Rh (Moyano et al., 2007). In 2013, we arranged two polyvinyl chloride polymer collars (20 cm diameter and 15 cm height) into the soil in each plot and selected one to set Nylon mesh bags with 40 cm deep × 25 cm diameter, and 33 μm aperture for root exclusion (Zhou et al., 2019). Rs and Rh were directly estimated on soil collar with and without root exclusion, while Ra was estimated by the difference value (*Ra = Rs - Rh*). Rs and its components were measured 12 across the whole growing season for all plots (It was planned to be measured every 10 days but adjusted for irresistible factors). The measurements were conducted between 10:00 and 14:00 BST (Beijing Summer Time) on sunny days by a portable infrared gas analyzer Li-8100 (LI-COR, Inc., Lincoln, NE, United States) with a stainless-steel jar. The jar lid was placed on each collar for 120 s to continuously record CO_2_ concentration at 1 s intervals, usually 15 to 30 s was required to reach a steady state between each measurement. The soil water content (SWC) at depth of 10 cm was measured at the time of Rs measurements by the external temperature sensor called T-type thermocouple (Li-COR, Inc., Lincoln, NE, United States) and water sensor called ML2X (LI-COR, Inc., Lincoln, NE, United States), respectively.

### Ecosystem CO_2_ exchange measurements

Gross primary productivity (GPP) was calculated by the difference between ecosystem respiration (RE) and net ecosystem exchange (NEE). NEE (with sunlight) and RE (with lightproof) were synchronously measured directly with Rs using a transparent chamber (50 cm × 50 cm × 50 cm) attached to an infrared gas analyzer (LI-840A, LI-COR Inc., Lincoln, NE, United States). In brief, CO_2_ concentration in the chamber was recorded every second until 120 s and the first and last 10 s were deleted. All flux measurements were conducted during the morning (9:00–11:30) on sunny days.

### Soil sample and soil property measurement

We collected three soil cores (3 cm in diameter and 10 cm in depth) and then mixed them into a composite fresh sample for each plot at the end of treatments. Each soil sample was sieved to ≤ 2 mm directly. The chloroform fumigation–extraction method was used to estimate soil microbial biomass carbon and nitrogen (MBC and MBN) (Vance et al., 1987). In brief, after being fumigated (10 g dry weight equivalent, fumigation for 24 h with ethanol-free CHCl_3_) and unfumigated, the fresh soil samples were extracted by shaking for 30 min in 60 ml of 0.5 M K_2_SO_4_. Then, the extracts were filtered and frozen at −20°C before being analyzed by dichromate digestion and Kjeldahl digestion. MBC and MBN were calculated as the difference between extractable carbon in the fumigated and nitrogen in the unfumigated samples using conversion factors of 0.38 and 0.45.

### Sensitivity of Rs to extreme drought

Ecologists have proposed a new definition of sensitivity to focus on the drought timing (Zhang et al., 2017; Liu et al., 2021), as the unit change of output per unit change of input in relative terms. To assess the sensitivity of soil respiration to drought seasonal events, the sensitivity was calculated as the relative change in response parameters of relative change in precipitation in the manipulation plots compared with the control plots, according to Eq. (1):


$$\mathrm{Sensitivity}=\frac{\left(X_{\mathrm{drought}}-X_{\mathrm{control}}\right)/X_{\mathrm{control}}}{\left({\mathrm{GSP}}_{\mathrm{control}}-{\mathrm{GSP}}_{\mathrm{drought}}\right)/{\mathrm{GSP}}_{\mathrm{control}}}$$

Where *X*_*drought*_ and *X*_*control*_ are mean Rs, Ra, or Rh across all drought and control plots, respectively. *GSP*_*drought*_ and *GSP*_*control*_ are the precipitation amounts in drought and control plots during the growing season. The sensitivity of drought seasonal events is expressed as the proportion of parameter response per precipitation change. Negative or positive values would indicate whether response parameters are suppressed (< 0) or promoted (> 0) by drought, while the absolute value is not of primary importance.

### Statistical analyses

Given the split-plot design, rainfall manipulation was restricted in the main plot and automatically implements the nesting of plant functional types. We used two-way ANOVA to test the effects of drought with seasonal timing, plant function types, and their interactions on seasonal mean SWC, GPP, Rs, Ra, and Rh and their sensitivities, MBC and MBN. Duncan’s multiple comparison (Duncan’s Multiple Range Test) was used to compare the mean difference of the above variables among these treatments in each plant function type. Homogeneity of variances and normally distributed errors had been met by *Levene* Test and Shapiro–Wilk test. If these assumptions were not satisfied, then the data were transformed using Box-Cox power. Linear regression was used to correlate Rs, Ra, and Rh with SWC in three PFT treatments, respectively. In addition, a path analysis was conducted to quantify the direct and indirect effects of extreme drought on Rs. Based on the previous research theories and hypothetical models (Dias et al., 2010; Burri et al., 2018; Dong et al., 2020), we established four main pathways, including the change of SWC caused by drought, biological factors (GPP and MBC), and sensitivity to explore the effects of drought on Rs. Especially, the validity of the model was tested using chi-square (χ2) tests, standardized root-mean-square residual (SRMR) index, root-mean-square error of approximation (RMSE) index, and goodness-of-fit index (CFI), and CFI close to one indicate a good model fit (Grace et al., 2010; Liu et al., 2017). All analyses were performed by R (4.0.0) and path analysis was performed using AMOS 24.0 (IBM; SPSS).

## Results

### Seasonal dynamics of soil moisture content

**FIGURE 1:**
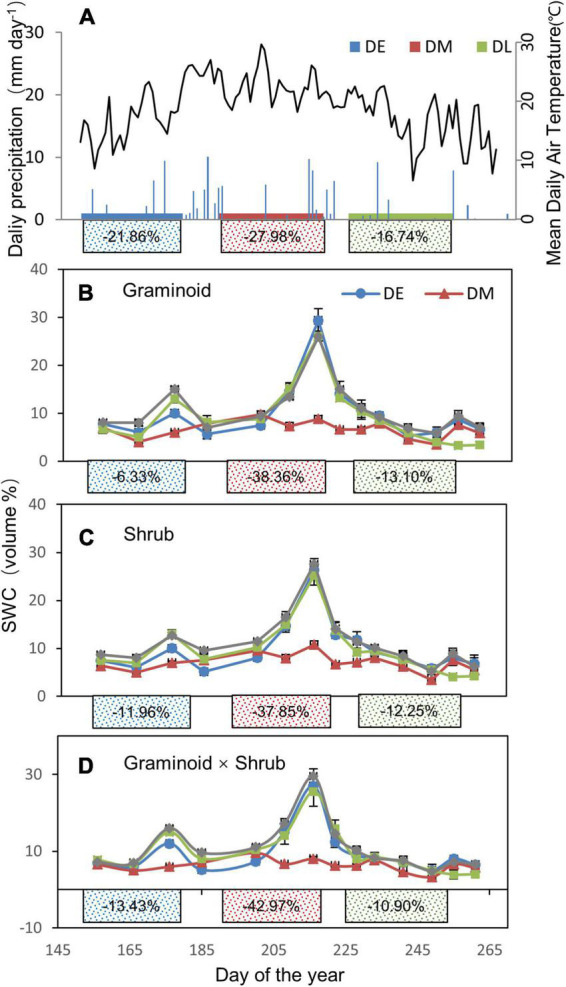
**(A)** Seasonal variations in daily precipitation (mm) and air temperature during the growing season of 2017. **(B–D)** Seasonal changes in soil water content (SWC, volume %) at 0 to 10 cm soil depth in three plant functional types (PFT): graminoids plots (G), shrub plots (S), and graminoid × shrub plots (GS) during the growing season in 2017, respectively. Shades **(A)** or lines **(B–D)** of blue, red, and green correspond to the drought treatment occurring in the early- (DE), mid- (DM), and late stage (DL) growing season, respectively. The solid rectangles indicate the occurring time of different extreme drought treatments (no effective rainfall 30 days’ interval). The numbers in the solid rectangles represent the mean decreased rainfall **(A)** and soil moisture **(B–D)** of each drought period compared with the corresponding ambient condition over the whole growing season, respectively.

Total growing season precipitation (GSP) was 130.8 mm in 2017, a 46.8% decrease compared with the long-term average (245.9 mm from 1953 to 2017). According to the probability density functions of growing season precipitation based on the ∼60-year data on this site, 2017 GSP was at the left of the 10th percentile (Supplementary Figure 1). The amounts of natural precipitation excluded were 28.6 mm, 36.6 mm, and 21.9 mm for the early-, mid-, and late stage drought treatment, respectively. There was a reduction of 21.86%, 27.98%, and 16.74% during the early-, mid-, and late stage drought treatments compared with the ambient treatment, respectively (Figure 1A).

Overall, extreme drought treatments significantly decreased the seasonal average SWC (F_3,24_ = 22.09, *P* < 0.01, Table 1). Over the whole growing season, in the Graminoid plots, SWC was decreased by 6.33, 38.36, and 13.10% in the early-, mid-, and late stage drought treatment, respectively (Figure 1B). Likewise, SWC was reduced by 11.96, 37.85, and 12.25% in the Shrub plots and 13.43%, 42.97%, and 10.90% in the Graminoid × Shrub plots in three seasonal drought treatments, respectively (Figures 1C–D). However, neither PFT treatments effect (F_2,24_ = 0.43, *P* = 0.66) nor the interaction between drought timings and PFT treatments on SWC were significant (F_6,24_ = 0.60, *P* = 0.72) (Table 1).

**TABLE 1:** Results of variance analysis of drought, plant function types (PFTs), and their interactive effects on soil water content (SWC), microbial biomass carbon (MBC) and nitrogen (MBN), gross primary productivity (GPP), soil respiration (Rs), autotrophic respiration (Ra), and heterotrophic respiration (Rh).

Source of variation	SWC			MBC			MBN			GPP			Rs			Ra			Rh		
	df	F	*P*	df	F	*P*	Df	F	*P*	df	F	*P*	df	F	*P*	df	F	*P*	df	F	*P*
Drought	3	22.09	**< 0.01**	3	12.76	**< 0.01**	3	16.97	**< 0.01**	3	9.88	**< 0.01**	3	60.34	**< 0.01**	3	9.55	**< 0.01**	3	58.20	**< 0.01**
PFT	2	0.43	0.66	2	0.47	0.63	2	0.83	0.45	2	8.38	**< 0.01**	2	7.89	**< 0.01**	2	0.17	0.73	2	13.77	**< 0.01**
Drought × PFT	6	0.60	0.72	6	1.05	0.42	6	1.48	0.23	6	3.66	**0.01**	6	3.38	**< 0.01**	6	0.83	0.55	6	1.72	**0.02**

Bold values indicated significant differences at p < 0.05.

### Response of soil respiration to droughts

**FIGURE 2:**
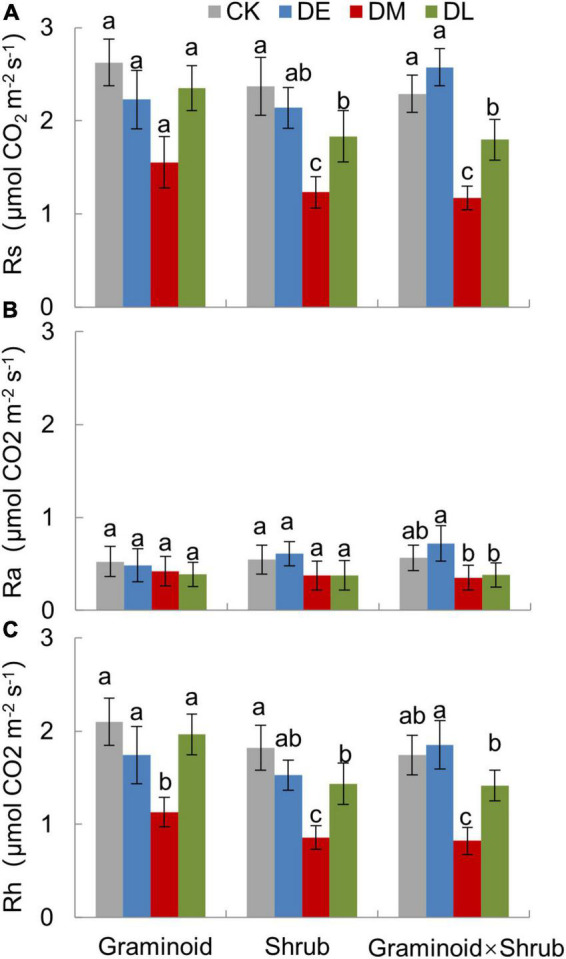
Growing season mean value of total soil respiration (Rs, **A**), autotrophic respiration (Ra, **B**), and heterotrophic respiration (Rh, **C**) under different drought timing treatments in three PFTs in 2017. Data are mean ± 1SE. Different letters indicate significant differences (*P* ≤ 0.05) among treatments.

Extreme drought significantly affected Rs (F_3,24_ = 60.34, *P* < 0.001) and Rh (F_3,24_ = 58.20, *P* < 0.001) across all three PFT treatments (Table 1 and Figure 2). Overall, mid-drought drastically reduced Rs and Rh during and after the treatment period in all three plant functional types, resulting in the largest reduction in Rs and Rh among the three droughts. In contrast, early drought had little effect on Rs and Rh. There were significant interactions between drought and PFT on Rs (*P* < 0.01) and Rh (*P* < 0.01), which mainly reflected that late drought suppressed Rs and Rh in shrub and combination communities but not in graminoid communities. Although drought also had significant effects on Ra (F_3,24_ = 9.55, *P* < 0.001), we did not find obvious differences between drought and ambient treatments in each PFT community.

### Sensitivity of soil respiration and its components to drought

**FIGURE 3:**
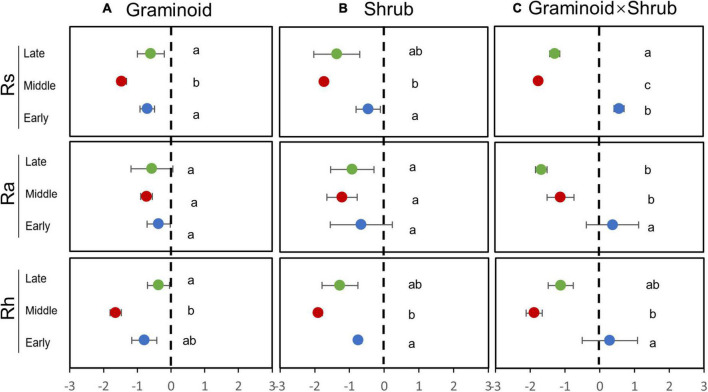
Sensitivity of Rs, Rh, and Ra responses to drought treatment in the G**(A)**, S**(B)**, and GS plots**(C)**. Sensitivity is a dimensionless parameter [Eq. (1)]. Different letters indicate significant differences (*P* ≤ 0.05) between treatments.

The seasonal extreme drought had significant effects on the sensitivity of Rs and its components (Table 2, all *P* ≤ 0.05). Similar to absolute values responses, Rs and Rh had the largest negative sensitivities to the mid-drought than the early- and late drought, while overall Ra had similar sensitivities to three droughts (Figure 3 and Table 3). Overall, Rh was more sensitive to droughts than Ra. PFT treatments had little effect on the sensitivity of Rs and its components, and a significant interaction effect between seasonal drought and PFT treatments occurred in the sensitivity of Rs (F_4,18_ = 2.93, *P* = 0.05), yet.

**TABLE 2:** Results of two-way ANOVA for the effects of droughts with seasonal timing, plant function types (PFTs), and their interaction on the sensitivity of soil respiration and its components.

	Rs			Ra			Rh		
	df	F	*P*	df	F	*P*	df	F	*P*
Drought	2	17.59	**< 0.01**	2	5.40	**0.02**	2	10.32	**< 0.01**
PFT	2	0.83	0.45	2	0.72	0.50	2	1.11	0.35
Drought × PFT	4	2.93	**0.05**	4	2..80	0.06	4	1.76	0.18

Bold values indicated significant differences at p < 0.05.

**FIGURE 4:**
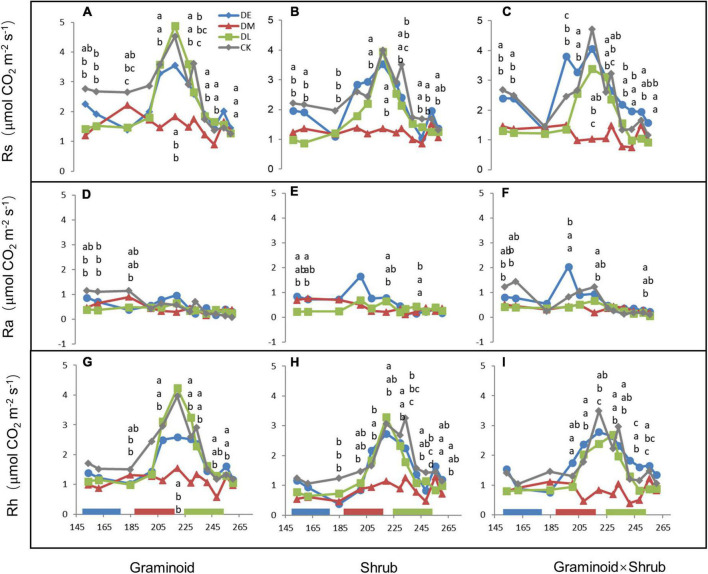
Seasonal dynamics of Rs **(A–C)**, Ra **(D–F)**, and Rh **(G–I)** in 2017, corresponding to PFTs of G, S, and GS, respectively. Letters, arranged in the same vertical order as the points they refer to, indicated significant differences (a, identical to controls). For clarity, only significant differences were depicted. Data are mean ± 1SE.

**TABLE 3:** Results from *t*-test of sensitivity of soil respiration and its components to drought imposed in early-, mid-, and late growing season, respectively.

		Graminoid						Shrub						Graminoid × Shrub					
	df	Early		Middle		Late		Early		Middle		Late		Early		Middle		Late	
		*t*	*P*	*t*	*P*	*t*	*P*	*t*	*P*	*t*	*P*	*t*	*P*	*t*	*P*	*t*	*P*	*t*	*P*
Soil respiration (Rs)	2	3.27	0.08	10.69	**< 0.01**	1.49	0.27	1.13	0.38	79.95	**< 0.01**	1.90	0.20	−4.02	**0.06**	23.58	**< 0.01**	8.65	**0.01**
Autotrophic respiration (Ra)	2	1.08	0.39	4.16	**0.05**	8.41	**0.01**	0.66	0.58	2.94	0.09	2.16	0.16	−1.51	0.27	4.15	**0.05**	12.88	**< 0.01**
Heterotrophic respiration (Rh)	2	2.12	0.17	9.85	**0.01**	1.13	0.38	7.42	**0.02**	16.89	**< 0.01**	2.38	0.14	−0.56	0.63	7.94	**0.02**	2.97	0.10

Bold values indicated significant differences at p < 0.05.

### Response of MBC, MBN, and GPP to droughts

**FIGURE 5:**
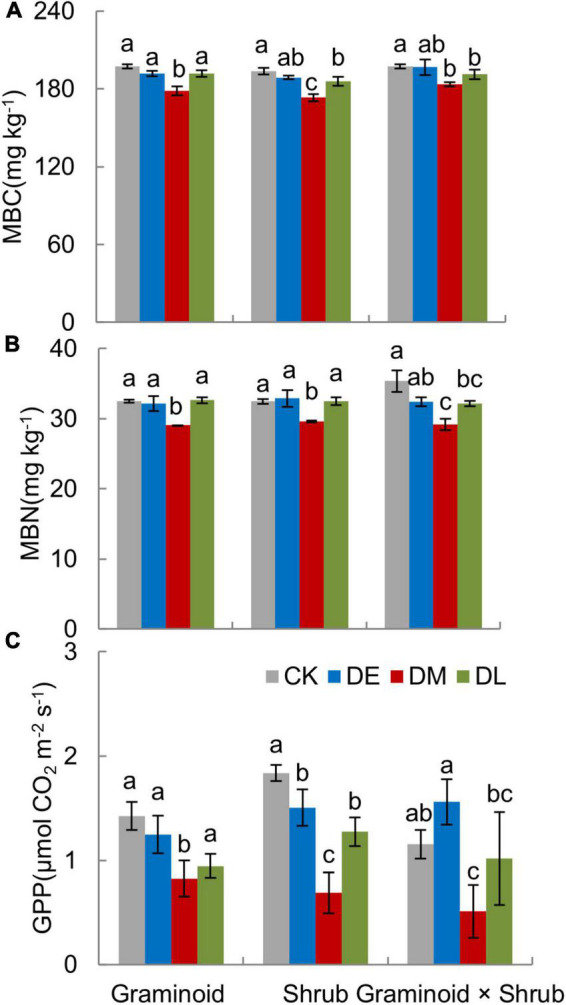
Growing season mean value of soil microbial biomass (MBC, **A**), microbial biomass nitrogen (MBN, **B**), and gross primary productivity (GPP, **C**) in three PFTs under different drought timing treatments in 2017. Data are mean ± 1SE. Different letters indicate significant differences (*P* ≤ 0.05) between treatments.

MBC, MBN, and GPP all showed the largest drop in response to mid-drought than the other two droughts in all PFT treatments (Figures 5A–C).

In the Graminoids plots, mid-stage drought reduced MBC, MBN, and GPP by 9.53, 10.54, and 42.28% compared with control treatments. Similarly, MBC, MBN, and GPP were reduced by 10.59, 8.82, and 62.61% in the Shrub plots and 7.09, 17.41, and 55.92% in the Graminoid × Shrub plots. GPP showed a decrease in early-stage drought, 12.50% reduction in the Graminoids, and 18.02% reduction in the Shrub plots but an insignificant increase in the Graminoid × Shrub plots (24.96%) (Figure 5C).

PFT treatments and interaction between drought and PFT also had a significant influence on GPP (both *P* < 0.01), which mainly reflected that late-drought suppressed GPP in shrub and combination communities but not in graminoid communities. Although interaction effect had no significant influence on MBC and MBN (Table 1, MBC: F_3,24_ = 0.47, *P* = 0.63; MBN: F_3,24_ = 0.83, *P* = 0.45), we also found MBC and MBN was suppressed by late-drought in shrub and combination communities.

### The influence of abiotic and biotic factors on soil respiration

**FIGURE 6:**
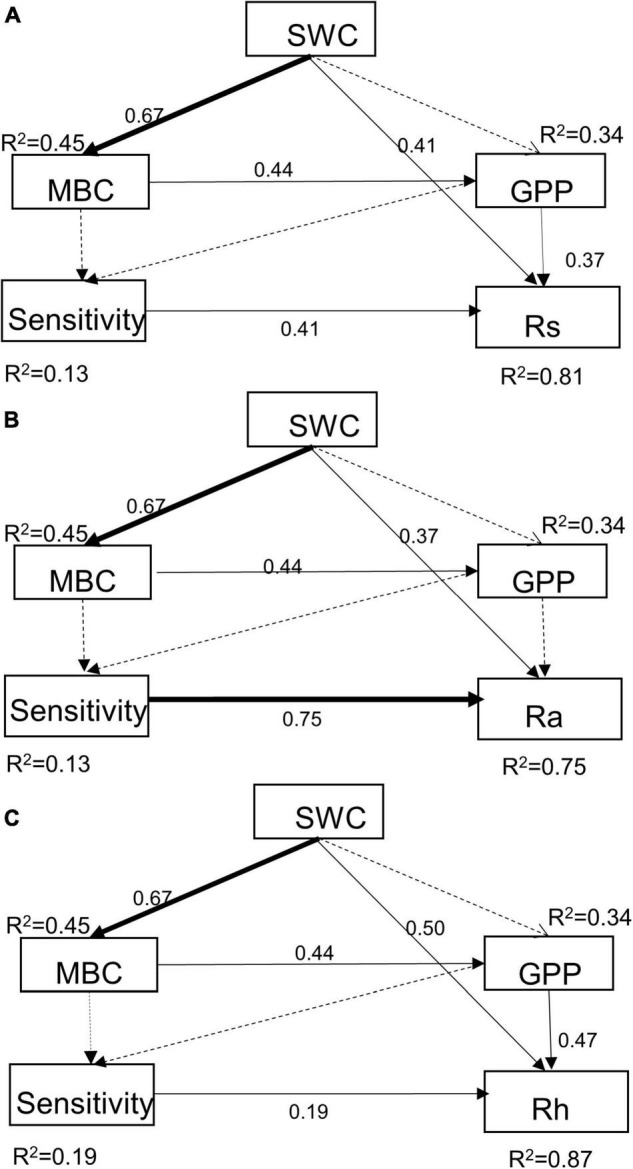
Path analysis of the effects of extreme drought joint with species composition changes in abiotic and biotic factors on Rs **(A)**, Ra **(B)**, and Rh **(C)**. For Rs pathway, χ2 = 0.43, *P* = 0.81, and df = 2, RMSEA < 0.01, AGFI = 0.99, CFI = 1.00; For Ra pathway, χ2 = 1.42, *P* = 0.49, and df = 2, RMSEA < 0.01, AGFI = 0.98, CFI = 1.00; For Rh pathway, χ2 = 1.87, *P* = 0.39, df = 2, RMSEA < 0.01, AGFI = 0.97, CFI = 1.00 (a high *P*-value associated with a χ2 test indicates a good fit between the model and the data, i.e., no significant discrepancies). Solid and dashed arrows represent significant (*P* ≤ 0.05) and non-significant relationships (*P* > 0.05). Values associated with solid arrows represent standardized path coefficients.

Results of path analysis showed that drought treatments reduced soil respiration mainly directly through SWC or indirectly through MBC and GPP (Figure 6). The changes in MBC, GPP, and sensitivity caused by soil moisture had positive effects on Rs, Ra, and Rh, some were not significant yet (Figure 6), and the influence of SWC was stronger in Rh than Ra with higher standardized regression weights than Rs (0.50 vs. 0.37). Moreover, the altered sensitivity induced by MBC and GPP had a significantly positive influence on Rs and its components. The direct positive relationships between the sensitivity and Rs, Ra, and Rh were quantified by path coefficients of 0.41, 0.75, and 0.19, respectively. In general, these approaches explained 81, 75, and 87% of the total variance in Rs, Ra, and Rh, respectively.

## Discussion

### Higher sensitivity of Rh to droughts than Ra

In our study, Ra and Rh showed asynchronous responses to drought events. Rs to drought was mainly determined by variations in seasonal mean Rh, nearly accounting for 75 to 85% of Rs (Figure 2), while Ra remained unchanged by and large during the growing season (Figure 4). Additionally, we found the sensitivity of Rs and Rh to mid-drought was lower than other droughts, no matter in which plant composition, while that is not obvious in Ra (Figure 3). Regardless of the functional types, the absolute value of Rh sensitivity was significantly higher than that of Ra in response to mid-drought and there was no significant difference to other droughts (Supplementary Figure 4), indicating higher sensitivity of Rh than Ra. The dominant role of Rh in response to drought stress was consistent with findings in past studies in the temperate steppe (Liu et al., 2019; Meng et al., 2021). This might be explained by the fact that the changes in water caused by drought mainly affect microbial activity directly, which has a stronger impact on Rh (Ru et al., 2018; Li et al., 2020). Compared with Ra, Rh exhibited a much stronger change in response to changing SWC, which led to the significant relationship between Rs and SWC (Supplementary Figure 2). Additionally, our result of path analysis indicated that process MBC and GPP regulated by SWC mainly regulated soil respiration in response to drought (Figure 6). Given that Rh was strongly related to microbial activity via decomposition of soil organic carbon (Moyano et al., 2013; Zhang et al., 2019a), the possible reason is that decreased microbial activity reduces the contact between substrates and extracellular enzymes involved in decomposition (Jassal et al., 2008; Kuzyakov and Gavrichkova, 2010; Weldmichael et al., 2020), and the decreased quantity and quality of beneficial microorganisms inhibits plant growth and GPP, leading to higher sensitivity of Rh to drought than Ra.

### Rs was larger affected by droughts in the middle growing season rather than in other seasons

In concert with our hypothesis, Rs was suppressed by mid- and late-drought regardless of different PFTs (Table 1, Figure 2), especially during drought period with a sharp decrease in soil water content (SWC) (Figure 4). Significant relationship between Rs and SWC in all PFTs provided further support for the above argument (Supplementary Figure 2). Our results accord with the largely accepted notion that soil water content is considered one of the most important factors affecting the temporal variation of soil respiration (Schimel et al., 2001; Shi et al., 2008; Ru et al., 2018), especially for arid and semi-arid ecosystem (Knapp et al., 2008; Liu L. et al., 2016; Felton et al., 2019).

Moreover, Rs was largely affected by droughts in the middle growing season rather than other seasons. Our results showed that Rs was more affected by middle drought than any other period (Figure 2). Sensitivity of Rs and its components to different drought treatments provided further support for the above argument (Figure 3), which directly compared the timing effects *per se*. Previous studies have shown that the middle of the growing season corresponds to the peak period of soil respiration and emission due to high temperatures (Lee et al., 2018; Li et al., 2019). In our study, the reduction in SWC during mid-drought treatment was 20% to 30% more than that during early drought treatment, while precipitation reduction was similar (Figure 1). This may stem from the fact that the high air temperature causes higher evapotranspiration and leads to greater water stress during mid drought. (Figure 1A). In previous results, the drought happening in the hot season caused larger water stress due to higher evapotranspiration than that in the relatively cool season which provided further support for the above argument (De Boeck et al., 2011).

Water stress reduced Rs mainly by inhibiting the microbes’ activity and the supply of photosynthetic substrates on a daily and seasonal time scale (Wang and Fang, 2009; Yan et al., 2011). Our result showed that mid-stage drought treatment had the strongest negative impacts on GPP and the proxy of microbial activity, MBC, leading to the largest reduction in this period (Figure 5), which was consistent with path analysis result that SWC reduced soil respiration by changing GPP and MBC (Figure 6). Interestingly, SWC had a more significant restriction on MBC than GPP (Figure 6). Previous studies considered low GPP during drought was due more to stomatal closure and consequently reduced photosynthesis in response to high vapor pressure deficit (Kolb et al., 2013). We speculate that extreme drought stress reduced Rs mainly through a negative effect on microbial activity and secondarily via suppression in substrate supply. This coincided with a meta-analysis study recently which suggested that the microbial activity showed high sensitivity to decreased precipitation and resulted in a large decrease in microbial biomass and respiration rate (Zhang and Zhang, 2016; Du et al., 2020). In addition, compared with the rapid and immediate effects of MBC, the climate extremes did not cause significant and rapid changes in the plant (Li et al., 2020).

### Plant functional types regulated responses of Rs to late droughts

The influence of PFTs on Rs and Rh response to drought depended on drought timing, and the regulation only occurred in the late drought treatment (Table 1, Figure 2). We unexpectedly observed that the growing season mean Rs and Rh was significantly reduced by late drought in Shrub and Graminoid × Shrub plots (Figure 2), which was consistent with the response of MBC and GPP to late drought (Figure 5). In contrast, the late drought did not significantly affect the MBC and GPP of the Graminoids community, so Rs was not significantly decreased in this community. Previous studies have found that plant species usually exhibit substantial differences in nutrient acquisition strategy, photosynthetic capacity, and litter quality, which ultimately affect both autotrophic and heterotrophic respiration (Du et al., 2018; Zhang et al., 2021). Besides, plant functional groups differ in their production of microbial diversity, such as AM hyphae, and will influence soil respiration by mediating AM fungal abundance (Emily, 2012; Johnson et al., 2015; Gui et al., 2018). It is likely that shrubs are deeper rooting which may allow them to access these soil water reservoirs (Jing et al., 2014; Hoover et al., 2017), and the presence of shrub functional groups will influence microbial activity and then modulate the response of soil respiration to drought.

And we speculate that the regulation of PFTs to drought stress depends on the intensity (Kuiper et al., 2014). There was no significant difference in Rs between the three communities in the mid- and early-stage drought treatment in the present study (Fig. 4), which may result in drought conditions in 2017. The drought stress was so severe that the regulation effects of the plant to maintain ecosystem stability was weakened (Kreyling et al., 2008) and not obvious, especially in the mid-stage of the growing season. While during an early-stage drought, the community has not recovered and the water use efficiency is similar among different functional types (Limousin et al., 2015), so the regulation difference of different plant functional types is little in the early growing season. However, we must recognize that our results are probably underestimating the effect of extreme droughts since treatment plots were compared to “control” plots that were also somehow drought stressed. Therefore, more data are needed to test the difference between normal years and dry years.

## Conclusion

In this study, we exposed a manipulative extreme-duration drought to three modeled plant function types (PFTs) during the early-, mid-, and late-stage of the growing season. Regardless of seasonal timing and plant functional type, Rh dominated negative responses of Rs to droughts, because low SWC induced reduction in MBC and GPP, while Ra overall unchanged under droughts in this semiarid grassland. However, the magnitude of the negative effects of droughts on Rs and Rh depended on seasonal timing and plant functional type. Interestingly, late drought reduced Rs and Rh in shrub and combination communities but not in graminoid communities. In summary, our results highlighted that Rs in response to droughts depended on both seasonal timing and plant functional type and that microbe and plant co-regulated Rs to droughts.

## Data availability statement

The raw data supporting the conclusions of this article will be made available by the authors, without undue reservation.

## Author contributions

YH conceived and designed the experiments. RQ, LL, ZZ, and FW performed the experiments. RQ and ZT analyzed the data. RQ and LL wrote the manuscript. All authors provided the editorial advice and contributed to the article and approved the submitted version.
