# Supplementary material for: Joint control of seasonal timing and plant function types on drought responses of soil respiration in a semiarid grassland
Source: Front Plant Sci. 2022 Aug 15;13:974418. doi: 10.3389/fpls.2022.974418 (PMC9421296; doi:10.3389/fpls.2022.974418)
Supplement: Supplementary file 1 [file Data_Sheet_1.DOCX]

Supplementary Material

# Supplementary Figures and Tables

## Supplementary Figures


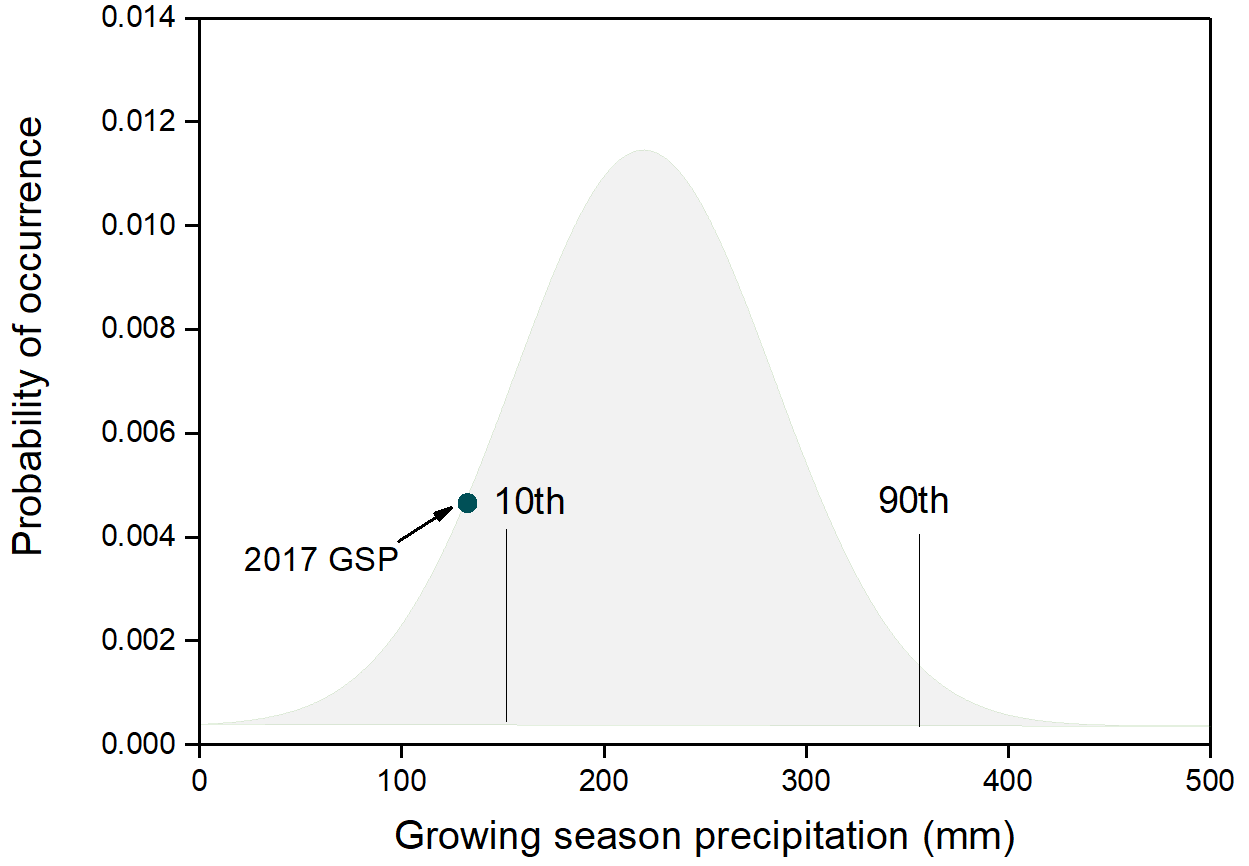


**Supplementary Figure S1.** Probability density functions of growing season precipitation of study site based on the ∼60-year data.


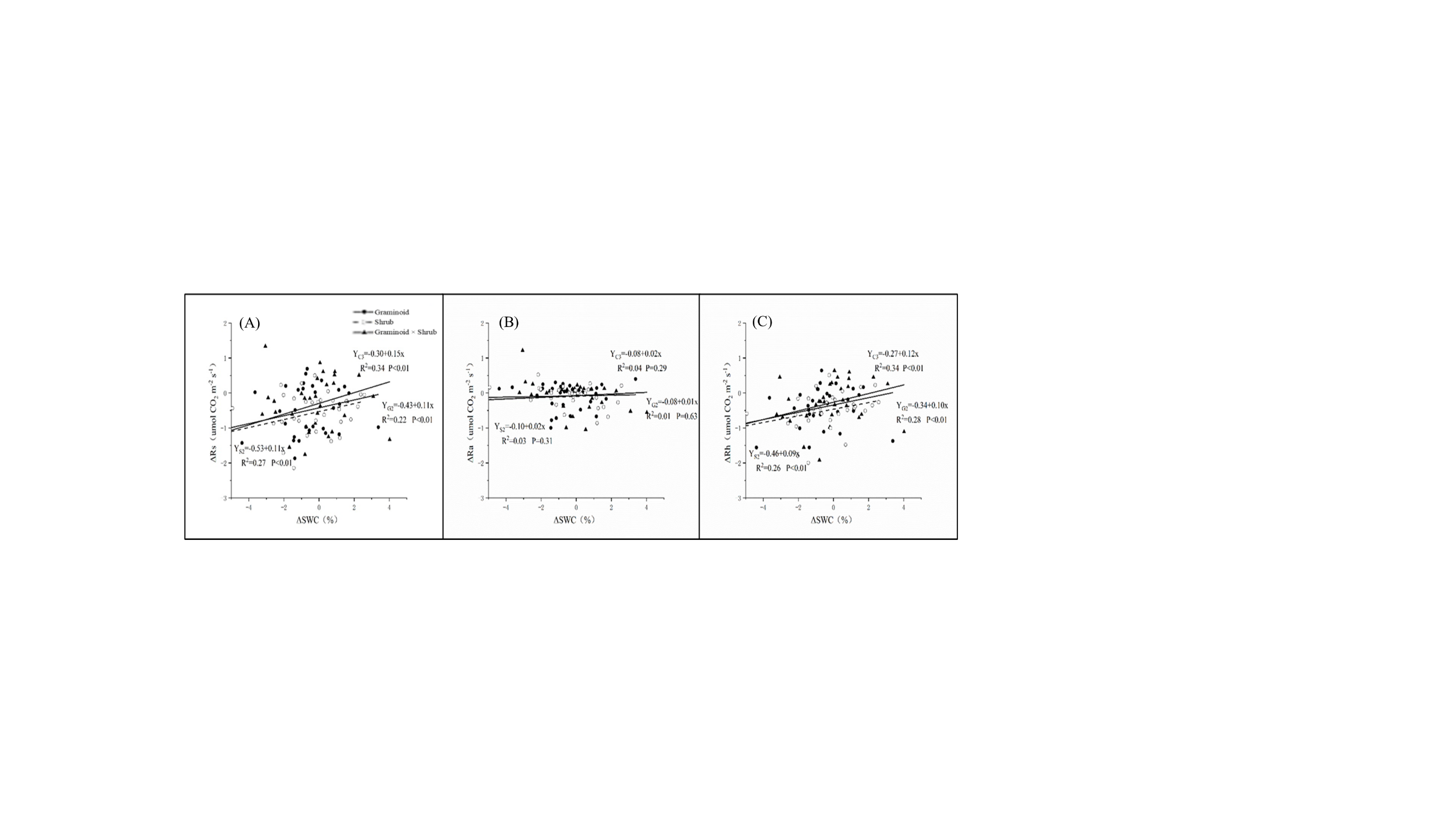


**Supplementary Figure S2.** Relationships of ΔRs, ΔRa and ΔRh (the difference in seasonal average Rs, Ra and Rh between the drought and ambient treatments) with the variations in soil water content (ΔSWC) (A-C) during three drought periods in the Graminoids (solid circle), Shrubs (hollow circle) and Graminoid×Shrub (solid triangle) plots, respectively.


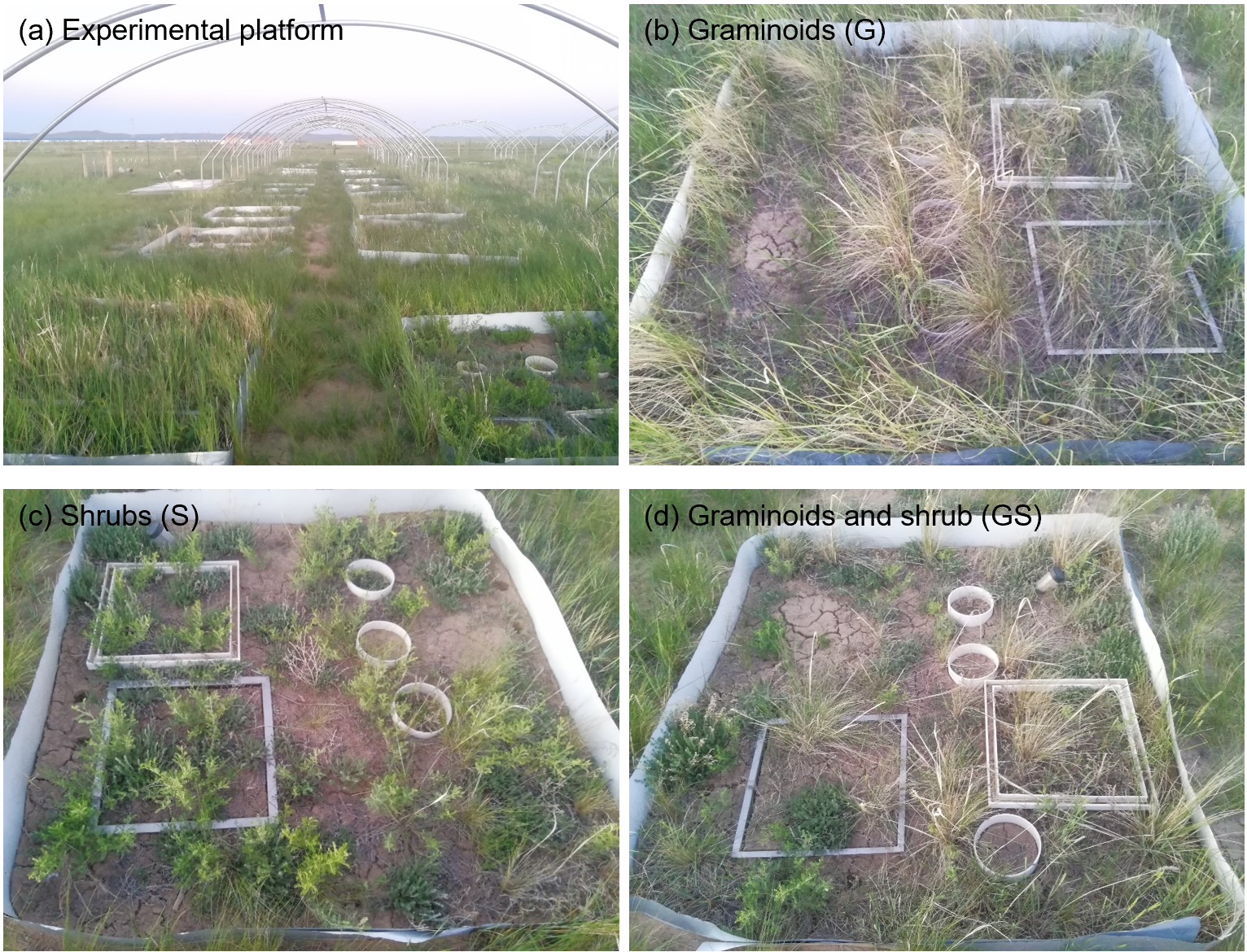


**Supplementary Figure S3.** Community physiognomy of the experiment platform and plant functional type treatments. The photos were taken in June, 2020.


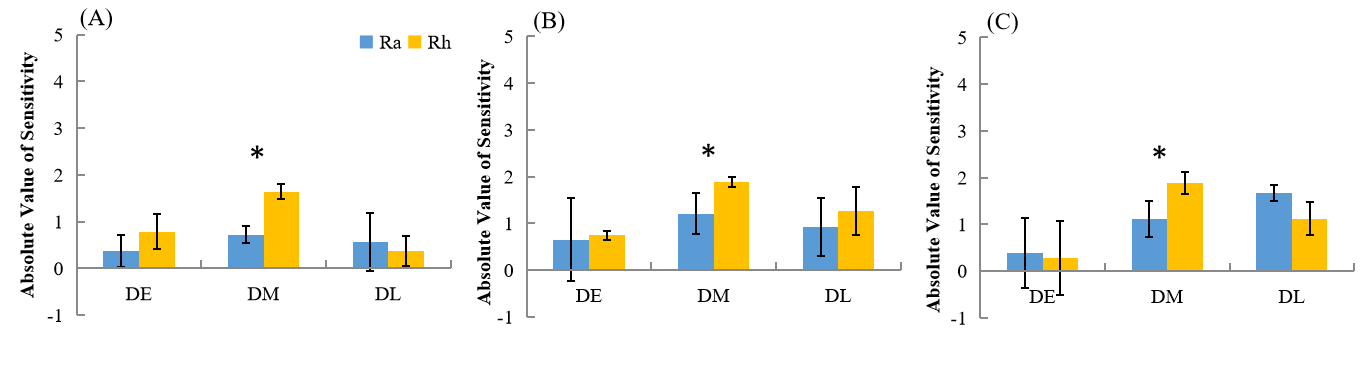


**Supplementary Figure S4.** Results of absolute sensitivity of Autotrophic respiration (Ra) and Heterotrophic respiration (Rh) to drought imposed in early-, mid- and late-growing season in Graminoid plots (A), Shrub plots (B) and Graminoid×Shrub plots (C), respectively. Data are absolute value of mean ±1SE。* indicates significant differences (P ≤ 0.05) between treatments
